# State-independent contextuality in classical light

**DOI:** 10.1038/s41598-019-51250-5

**Published:** 2019-11-19

**Authors:** Tao Li, Qiang Zeng, Xiong Zhang, Tian Chen, Xiangdong Zhang

**Affiliations:** 1 Key Laboratory of Advanced Optoelectronic Quantum Architecture and Measurements of Ministry of Education, School of Physics, Beijing Institute of Technology, Beijing, 100081 China; 2 College of Optical and Electronic Technology, China Jiliang University, Hangzhou, 310018 China

**Keywords:** Micro-optics, Micro-optics

## Abstract

State-independent contextuality is a fundamental phenomenon in quantum mechanics, which has been demonstrated experimentally in different systems in recent years. Here we show that such contextuality can also be simulated in classical optical systems. Using path and polarization degrees of freedom of classical optics fields, we have constructed the classical trit (cetrit), here the term ‘cetrit’ is the classical counterpart of a qutrit in quantum systems. Furthermore, in classical optical systems we have simulated the violations of several Yu-Oh-like noncontextual inequalities in a state-independent manner by implementing the projection measurements. Our results not only provide new physical insights into the contextuality and also show the application prospects of the concepts developed recently in quantum information science to classical optical systems and optical information processes.

## Introduction

Quantum contextuality is an important feature of quantum mechanics, and shows the discrepancy between quantum phenomenon and classical cognition. For a classical measure, the object measured has a determined value regardless of how the measurement is implemented. In contextuality case, the value of observable depends on its measurement together with its compatible observable. This phenomenon exhibits the violation of noncontextual inequality. In recent years, the contextuality has attracted great attention, because the investigation on contextuality is not only related to fundamental physics^1,2^, but also to practical quantum information processes^3^.

The quantum contextuality was first proposed by Kochen and Specker (KS)^4,5^ and Bell^6^ in 1960s. The original theory needs 117 vectors in dimension d = 3 (in fact, there are 192 vectors, some vectors that share one edge have been dropped for the figure simpler^7^), thus it is complex and nearly impossible to demonstrate experimentally. Afterwards the theory is simplified by many researchers^7–12^. Klyachko, Can, Binicioglu and Shumovsky (KCBS) proposed an inequality that involves 5 variables in three-level system^13^, but this inequality is state-dependent. Yu and Oh proposed a state-independent contextual inequality that just 13 variables and 24 correlation pairs are involved^14^. Pavičić pointed out that Yu-Oh’s scenario possesses 25 variables (corresponding to vectors containing in 16 edges), of which 12 (whose corresponding vectors share only edge) were omitted^15^. Pavičić also pointed out that the Yu-Oh’ set is not the KS set and is a new kind of contextuality^15^. It has been proven that in three-level system Yu-Oh’s scheme possesses the least variables for a state-independent contextuality. Subsequently, the improved and optimal forms of Yu-Oh inequality have been given^16,17^. The various contextual theories have been testified experimentally, for instance, in photon^18–26^, neutron^27,28^, trapped ion^29,30^, nuclear magnetic resonance^31^, and superconducting circuit systems^32^.

On the other hand, recent investigations have also shown that many quantum phenomena, which are considered as the unique properties of quantum system, can also be simulated in classical systems, such as the violations of Clauser-Horne-Shimony-Holt (CHSH) Bell inequality in classical wave systems^33–43^, the Hardy’s thought experiment in classical light^44^, violating the Leggett-Garg inequality in classical optical systems^45^, the violation of Mermin’s inequality in classical nonseparability systems^46^, and so on. Recently, the state-dependent contextuality involving 5 variables has been studied in classical optical systems, and violations of the Klyachko-Can-Binicioglu-Shumovski (KCBS) inequality and its geometrical form (Wright’s inequality) have been demonstrated experimentally^47^. The two-bit state-independent contextuality has also been explored in classical microwave systems^48^. However, the state-independent contextualities in three-level system (Yu-Oh inequality^14^, its improved form^16^, and its optimal forms^17^) have not been discussed in classical systems. The problem is whether the violations of these similar contextual inequalities can be simulated in classical optical systems?

In this work, we use the path and polarization degrees of freedom of classical optical beam to establish the classical trit (cetrit, corresponding to the qutrit in quantum system), and explore the violations of several noncontextual inequalities in a state-independent manner by implementing the projection measurements. By measuring the intensities at the output ports, which then are normalized by the total intensity, the average values of the observables and observable pairs are obtained. Furthermore, the violations of original Yu-Oh inequality, its improved and optimal forms have been observed experimentally in classical optical systems. Our study not only provokes deep thought on the contextuality in the classical and quantum systems, but also enriches the conceptual issues in optical coherence theory and suggests potential applications in the wave information process.

## Results and Discussions

### Theoretical description on state-independent contextuality in classical optical systems

According to refs^14,16,17^, the state-independent contextuality are presented in three-level system. In the three-level system, there are qutrit and 13 observables. The 13 observables *A*_*i*_ correspond to 13 unit vectors *a*_*i*_ (*i* = 1, ..., 13), and these unit vectors are expressed as

1 $$\begin{array}{rcl}{a}_{1}({h}_{1}) & = & \frac{1}{\sqrt{3}}(\,-\,1,1,1),\,{a}_{2}({h}_{2})=\frac{1}{\sqrt{3}}(1,-\,1,1),\,{a}_{3}({h}_{3})=\frac{1}{\sqrt{3}}(1,1,-\,1),\,{a}_{4}({h}_{0})=\frac{1}{\sqrt{3}}(1,1,1),\\ {a}_{5,6}({y}_{1}^{\pm }) & = & \frac{1}{\sqrt{2}}(0,1,\pm \,1),\,{a}_{7,8}({y}_{2}^{\pm })=\frac{1}{\sqrt{2}}(1,0,\pm \,1),\,{a}_{9,10}({y}_{3}^{\pm })=\frac{1}{\sqrt{2}}(1,\pm \,1,0),\\ {a}_{11}({z}_{1}) & = & (1,0,0),\,{a}_{12}({z}_{2})=(0,1,0),\,{a}_{13}({z}_{3})=(0,0,1),\end{array}$$

where the symbols behind *a*_*i*_ in the parentheses are the expressions of unit vectors in various inequality forms^14,16,17^. For the 13 operators $|{a}_{i}\rangle \langle {a}_{i}|$, their eigenvalues are 0 or 1. Here we make a transform, which is ${A}_{i}\,=\,I-2|{a}_{i}\rangle \langle {a}_{i}|$, where *I* is the identity matrix, so the observables *A*_*i*_ have two eigenvalues +1 or −1. If the measurement outcomes of *A*_*i*_ (or *A*_*j*_) are the noncontextual value +1 or −1, the original Yu-Oh inequality^14^ is obtained. It is listed in the middle column of the second row in Table 1, where Γ_*i,j*_ are the coefficients. If the observables *A*_*i*_ and *A*_*j*_ are compatible (the corresponding vectors are orthogonal), the value of Γ_*i,j*_ is 1. If *A*_*i*_ and *A*_*j*_ are not compatible, the value of Γ_*i,j*_ is 0. The Yu-Oh inequality can be obtained by the exhaustive check of the value +1 or −1 of *A*_*i*_ and *A*_*j*_ or an elegant analytic demonstration^14^. But in the case of quantum mechanics, for any qutrit state, the Yu-Oh inequality is violated^14^, namely $\mathop{\sum }\limits_{i=1}^{13}\langle {A}_{i}\rangle -\frac{1}{4}\mathop{\sum }\limits_{i=1}^{13}\mathop{\sum }\limits_{j=1,j\ne i}^{13}{\Gamma }_{i,j}\langle {A}_{i}{A}_{j}\rangle =\frac{25}{3}$, where $\langle {A}_{i}\rangle$ denotes the mean value of the operator *A*_*i*_ and $\langle {A}_{i}{A}_{j}\rangle$ denotes the mean value of the produce *A*_*i*_ *A*_*j*_ of measurement outcome. In Yu-Oh’s scenario, the inequality can be violated through 13 observables and 24 observable pairs^14^, so that the 12 additional observables corresponding to the 12 discarded vectors^15^, need not be involved. The inequality and its quantum violation are all listed in the second row of Table 1.

**Table 1:** The various noncontextuality inequalities and their quantum violations (refs^14,16,17^).

Contextuality forms	Inequalities	Quantum violations
Original Yu-Oh form^14^	$\mathop{\sum }\limits_{i=1}^{13}\langle {A}_{i}\rangle -\frac{1}{4}\mathop{\sum }\limits_{i=1}^{13}\mathop{\sum }\limits_{j=1,j\ne i}^{13}{\Gamma }_{i,j}\langle {A}_{i}{A}_{j}\rangle \le 8$	$\frac{25}{3}$
Improved form^16^	$\frac{1}{2}(\mathop{\sum }\limits_{i=1}^{4}\langle {A}_{i}\rangle -\mathop{\sum }\limits_{i=1}^{4}\mathop{\sum }\limits_{j=5}^{10}{\Gamma }_{i,j}\langle {A}_{i}{A}_{j}\rangle )+\mathop{\sum }\limits_{k=5}^{13}\langle {A}_{k}\rangle -\mathop{\sum }\limits_{m=5}^{12}\mathop{\sum }\limits_{n > m}^{13}{\Gamma }_{m,n}\langle {A}_{m}{A}_{n}\rangle \le 9$	$\frac{29}{3}$
Optimal form opt_2_^17^	$$\begin{array}{c}\mathop{\sum }\limits_{i=1}^{6}\langle {A}_{i}\rangle +2\mathop{\sum }\limits_{j=7}^{11}\langle {A}_{j}\rangle +3\mathop{\sum }\limits_{k=12}^{13}\langle {A}_{k}\rangle -\mathop{\sum }\limits_{i=1}^{4}\mathop{\sum }\limits_{j=5}^{10}{\Gamma }_{i,j}\langle {A}_{i}{A}_{j}\rangle -\mathop{\sum }\limits_{m=5}^{6}\langle {A}_{m}{A}_{11}\rangle \\ \,-2\mathop{\sum }\limits_{m=7}^{10}\mathop{\sum }\limits_{n > m}^{13}{\Gamma }_{m,n}\langle {A}_{m}{A}_{n}\rangle -\mathop{\sum }\limits_{n=12}^{13}\langle {A}_{11}{A}_{n}\rangle -2\langle {A}_{12}{A}_{13}\rangle \le 16\end{array}$$	$\frac{52}{3}$
Optimal form opt_3_^17^	$$\begin{array}{c}2\mathop{\sum }\limits_{i=1}^{4}\langle {A}_{i}\rangle +\mathop{\sum }\limits_{j=5}^{13}\langle {A}_{j}\rangle -2\mathop{\sum }\limits_{i=1}^{4}\mathop{\sum }\limits_{j=5}^{10}{\Gamma }_{i,j}\langle {A}_{i}{A}_{j}\rangle -\mathop{\sum }\limits_{m=5}^{10}\mathop{\sum }\limits_{n > m}^{13}{\Gamma }_{m,n}\langle {A}_{m}{A}_{n}\rangle \\ \,-2\mathop{\sum }\limits_{m=11}^{12}\mathop{\sum }\limits_{n > m}^{13}\langle {A}_{m}{A}_{n}\rangle -3(\langle {A}_{5}{A}_{6}{A}_{11}\rangle +\langle {A}_{7}{A}_{8}{A}_{12}\rangle +\langle {A}_{9}{A}_{10}{A}_{13}\rangle )\le 25\end{array}$$	$\frac{83}{3}$

In ref.^16^, the original Yu-Oh inequality is improved and its coefficients are changed, and a new inequality obtained is listed in middle column of the third row in Table 1. Here the requirement for Γ_*m,n*_ is the same as the requirement for Γ_*i,j*_, and its value corresponds to the compatible relation between *A*_*m*_ and *A*_*n*_. In the case of quantum mechanics^16^, for any qutrit state the result $\frac{29}{3}$ can be obtained, and the inequality shows the violation.

In addition, Kleinmann *et al*. proposed two other optimal inequalities opt_2_ and opt_3_ ^17^ corresponding to the original Yu-Oh inequality, but the coefficients before the observables are given in tabular form in original literature, which are not intuitive. After our sorting out, the two inequalities are showed in the middle columns of the fourth and fifth row in Table 1. However, their quantum violations are $\frac{52}{3}$ and $\frac{83}{3}$, respectively^17^, these are also listed in the Table 1.

The above descriptions are about the original Yu-Oh inequality, its improved and optimal forms in quantum mechanics. Now we give the corresponding descriptions in classical optical systems. We rewrite the corresponding unit vectors ${A^{\prime} }_{i}$ in the classical optical system as

2 $$\begin{array}{rcl}{a^{\prime} }_{1}({h^{\prime} }_{1}) & = & \frac{1}{\sqrt{3}}(\,-\,1,1,1),\,{a^{\prime} }_{2}({h^{\prime} }_{2})=\frac{1}{\sqrt{3}}(1,-\,1,1),\,{a^{\prime} }_{3}({h^{\prime} }_{3})=\frac{1}{\sqrt{3}}(1,1,-\,1),\\ {a^{\prime} }_{4}({h^{\prime} }_{0}) & = & \frac{1}{\sqrt{3}}(1,1,1),\,{a^{\prime} }_{5,6}({y^{\prime} }_{1}^{\pm })=\frac{1}{\sqrt{2}}(0,1,\pm \,1),\,{a^{\prime} }_{7,8}({y^{\prime} }_{2}^{\pm })=\frac{1}{\sqrt{2}}(1,0,\pm \,1),\\ {a^{\prime} }_{9,10}({y^{\prime} }_{3}^{\pm }) & = & \frac{1}{\sqrt{2}}(1,\pm \,1,0),\,{a^{\prime} }_{11}({z^{\prime} }_{1})=(1,0,0),\,{a^{\prime} }_{12}({z^{\prime} }_{2})=(0,1,0),\,{a^{\prime} }_{13}({z^{\prime} }_{3})=(0,0,1).\end{array}$$

**Figure 1:**
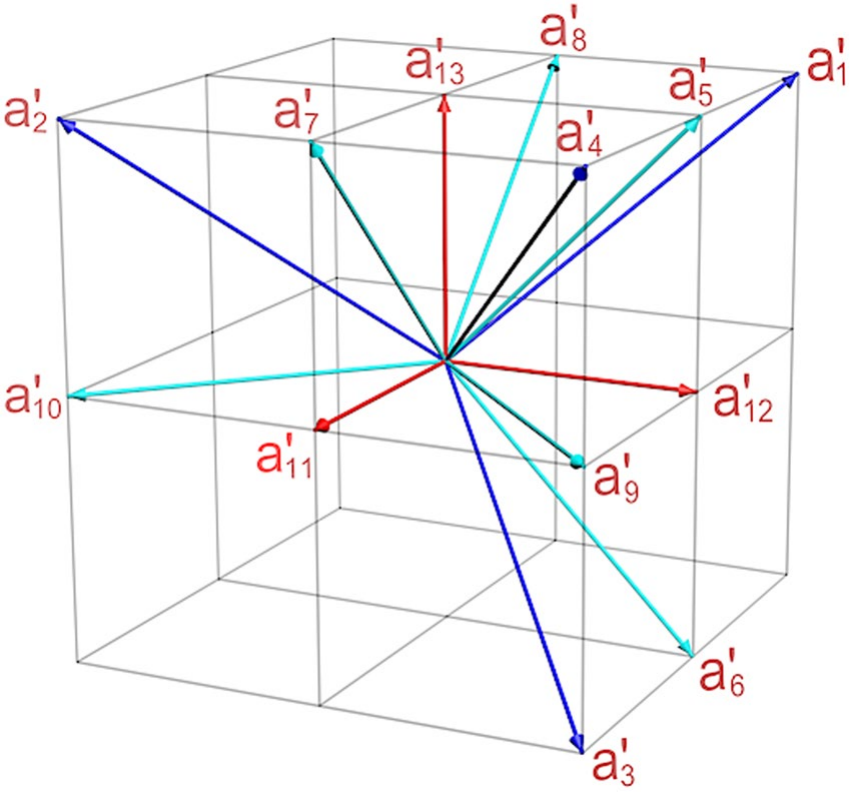
The illustration of the 13 unit vectors in classical optical system. The 13 unit vectors correspond to 13 observables. In order to testify the state independent contextuality, the 13 observables and their correlation pairs need to be measured in the experiment.

These unit vectors ${a^{\prime} }_{i}$ are showed in Fig. 1, and the 13 corresponding operators are expressed as $|{a^{\prime} }_{i})({a^{\prime} }_{i}|$. Here a slightly modified version of the familiar bra-ket notation of quantum mechanics is taken to express the vectors in classical optical fields. Similarly, the dichotomic observables are ${A^{\prime} }_{i}=I-2|{a^{\prime} }_{i})({a^{\prime} }_{i}|$, and the eigenvalues of ${A^{\prime} }_{i}$ are +1 or −1.

As shown in Eq. (2), the expressions of the vectors in classical case are similar to the vectors in quantum case. For any cetrit state |*χ*), we can write

3 $$|\chi )={E}_{0}|{\overrightarrow{{\rm{e}}}}_{0})+{E}_{1}|{\overrightarrow{{\rm{e}}}}_{1})+{E}_{2}|{\overrightarrow{{\rm{e}}}}_{2}),$$

where *E*_0_, *E*_1_ and *E*_2_ are the amplitudes of the classical optical fields, and $|{\overrightarrow{{\rm{e}}}}_{0})$, $|{\overrightarrow{{\rm{e}}}}_{1})$ and $|{\overrightarrow{{\rm{e}}}}_{2})$ are the cetrit bases corresponding to quantum bases $|0\rangle$, $|1\rangle$ and $|2\rangle$. In order to give the classical analogy of Yu-Oh original form, the corresponding operators in the left-hand side of Yu-Oh inequality are expressed as the multiples of identity matrix^14^, namely $\mathop{\sum }\limits_{i=1}^{13}{\hat{A^{\prime} }}_{i}-\frac{1}{4}\mathop{\sum }\limits_{i=1}^{13}\mathop{\sum }\limits_{j=1,j\ne i}^{13}{\Gamma }_{i,j}{\hat{A^{\prime} }}_{i}{\hat{A^{\prime} }}_{j}=\frac{25}{3}I$. When these operators corresponding to the observables project onto any cetrit states, the average values of the operators can be obtained, namely, $\mathop{\sum }\limits_{i=1}^{13}\overline{{A^{\prime} }_{i}}-\frac{1}{4}\mathop{\sum }\limits_{i=1}^{13}\mathop{\sum }\limits_{j=1,j\ne i}^{13}{\Gamma }_{i,j}\overline{{A^{\prime} }_{i}{A^{\prime} }_{j}}=(\chi |\tfrac{25}{3}I|\chi )=\tfrac{25}{3}({E}_{0}^{2}\,+\,{E}_{1}^{2}\,+\,{E}_{2}^{2})$, where $\overline{{A^{\prime} }_{i}}$ and $\overline{{A^{\prime} }_{i}{A^{\prime} }_{j}}$ denote the average values of the observable ${A^{\prime} }_{i}$ and ${A^{\prime} }_{i}{A^{\prime} }_{j}$ in classical case, respectively. When *E*_0_, *E*_1_ and *E*_2_ are normalized, the average value is $\tfrac{25}{3}$, namely

4 $$\mathop{\sum }\limits_{i=1}^{13}\overline{{A^{\prime} }_{i}}-\frac{1}{4}\mathop{\sum }\limits_{i=1}^{13}\mathop{\sum }\limits_{j=1,j\ne i}^{13}{\Gamma }_{i,j}\overline{{A^{\prime} }_{i}{A^{\prime} }_{j}}=\frac{25}{3} > 8,$$

The noncontextuality inequalities are violated.

Similarly, for the improved form in ref.^16^ and the optimal forms opt_2_ and opt_3_ in ref.^17^, the operators in the left-hand side of inequalities in Table 1 are also expressed as the multiples of the identity matrix, namely, $\frac{29}{3}I$, $\frac{52}{3}I$ and $\frac{83}{3}I$. When the operators project onto any cetrit states, the inequalities are violated. That is

5 $$\frac{1}{2}(\mathop{\sum }\limits_{i=1}^{4}\overline{{A^{\prime} }_{i}}-\mathop{\sum }\limits_{i=1}^{4}\mathop{\sum }\limits_{j=5}^{10}{\Gamma }_{i,j}\overline{{A^{\prime} }_{i}{A^{\prime} }_{j}})+\mathop{\sum }\limits_{k=5}^{13}\overline{{A^{\prime} }_{k}}-\mathop{\sum }\limits_{m=5}^{12}\mathop{\sum }\limits_{n > m}^{13}{\Gamma }_{m,n}\overline{{A^{\prime} }_{m}{A^{\prime} }_{n}}=\frac{29}{3} > \mathrm{9,}$$

6 $$\begin{array}{c}\mathop{\sum }\limits_{i=1}^{6}\overline{{A^{\prime} }_{i}}+2\mathop{\sum }\limits_{j=7}^{11}\overline{{A^{\prime} }_{j}}+3\mathop{\sum }\limits_{k=12}^{13}\overline{{A^{\prime} }_{k}}-\mathop{\sum }\limits_{i=1}^{4}\mathop{\sum }\limits_{j=5}^{10}{\Gamma }_{i,j}\overline{{A^{\prime} }_{i}{A^{\prime} }_{j}}-\mathop{\sum }\limits_{m=5}^{6}\overline{{A^{\prime} }_{m}{A^{\prime} }_{11}}\\ \,\,\,\,\,\,-\,2\mathop{\sum }\limits_{m=7}^{10}\mathop{\sum }\limits_{n > m}^{13}{\Gamma }_{m,n}\overline{{A^{\prime} }_{m}{A^{\prime} }_{n}}-\mathop{\sum }\limits_{n=12}^{13}\overline{{A^{\prime} }_{11}{A^{\prime} }_{n}}-2\overline{{A^{\prime} }_{12}{A^{\prime} }_{13}}=\frac{52}{3} > 16,\end{array}$$

7 $$\begin{array}{c}2\mathop{\sum }\limits_{i=1}^{4}\overline{{A^{\prime} }_{i}}+\mathop{\sum }\limits_{j=5}^{13}\overline{{A^{\prime} }_{j}}-2\mathop{\sum }\limits_{i=1}^{4}\mathop{\sum }\limits_{j=5}^{10}{\Gamma }_{i,j}\overline{{A^{\prime} }_{i}{A^{\prime} }_{j}}-\mathop{\sum }\limits_{m=5}^{10}\mathop{\sum }\limits_{n > m}^{13}{\Gamma }_{m,n}\overline{{A^{\prime} }_{m}{A^{\prime} }_{n}}\\ \,\,-\,2\mathop{\sum }\limits_{m=11}^{12}\mathop{\sum }\limits_{n > m}^{13}\overline{{A^{\prime} }_{m}{A^{\prime} }_{n}}-3(\overline{{A^{\prime} }_{5}{A^{\prime} }_{6}{A^{\prime} }_{11}}+\overline{{A^{\prime} }_{7}{A^{\prime} }_{8}{A^{\prime} }_{12}}+\overline{{A^{\prime} }_{9}{A^{\prime} }_{10}{A^{\prime} }_{13}})=\frac{83}{3} > 25.\end{array}$$

Similarly, the symbols with the horizontal lines in Eqs (5–7) denote the average values of the observables. In the following, we test experimentally the above inequalities in classical light systems.

### Experimental demonstration of state-independent contextuality for the original Yu-Oh inequality in classical optics systems

**Figure 2:**
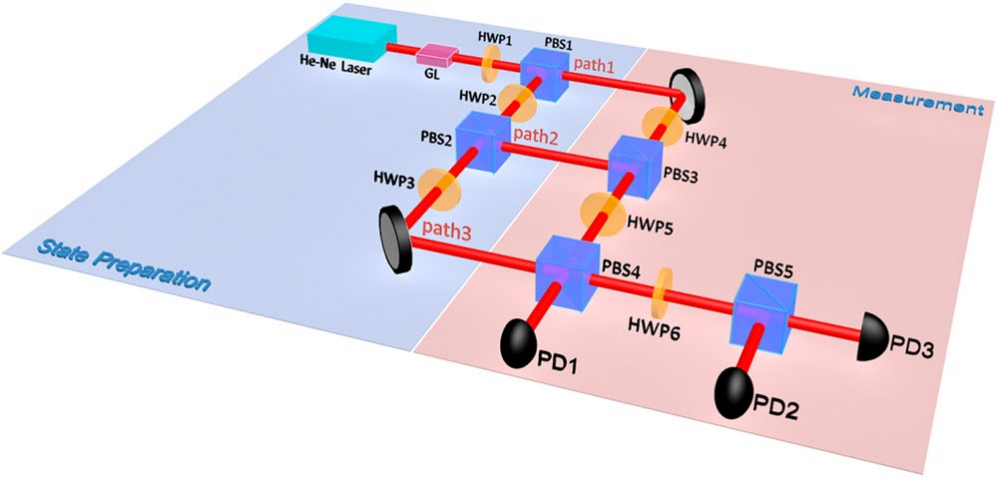
The diagram of experimental setup. The experiment consists of two stages, the state preparation stage and the measurement stage. GL: Glan lens. HWP(*i*): half wave plate (*i* = 1, …, 6). PBS(*i*): polarizing beam splitter (*i* = 1, …, 5). PD(*i*): photoelectric detector (*i* = 1, …, 3). The angles of HWP3 and 4 are set as 0° for path-length compensation.

In this section, we describe the experimental demonstration of state-independent contextuality for the original Yu-Oh inequality. In order to test the state-independent contextuality in classical optical systems, the constructed experimental setup is shown in Fig. 2. It is divided into two stages: state preparation and measurement. In the state preparation stage, because it is state-independent contextuality, several different cetrit input states need to be prepared. The laser beam from He-Ne laser transmits through the polarizing beam splitter (PBS) (is not shown in Fig. 2) and the Glan lens, and then the horizontal polarization beam can be obtained. There the central wavelength of He-Ne laser is 633 nm. After the horizontal polarization beam transmits through the half wave plate 1 (HWP1) and a PBS1, it is divided into two beams. One of the beams transmits through a HWP2 and a PBS2, thereupon three beams of light are obtained. The horizontal polarization field in the first path is coded as $|{\overrightarrow{{\rm{e}}}}_{0})$, the vertical polarization field in the second path is coded as $|{\overrightarrow{{\rm{e}}}}_{1})$, and the horizontal polarization field in the third path is coded as $|{\overrightarrow{{\rm{e}}}}_{2})$. The polarization fields in the three paths are taken as the basis vectors to constitute the input cetrit states. With tuning the angles of HWP1 and HWP2, any composition of the optical fields for the three paths can be obtained, thus the desired input state can be prepared. Here seven different cetrit input states are prepared. The seven input states and the setting angles of HWP1 and HWP2 for the input state preparations are listed in the table (see Methods section: The setting angles of HWPs for the different input state preparations).

In the measurement stage, the projection measurement method is adopted. The input state projects onto the eigenstates of the observable, and the probabilities of eigenvalues can be gotten. When we sum the product of each eigenvalue and its probability, the average value of the observable can be calculated. This is $\overline{M}=(\chi |\hat{M}|\chi )=(\chi |[\sum _{i}{m}_{i}|{m}_{i})({m}_{i}|]|\chi )={\sum _{i}{m}_{i}|(\chi |{m}_{i})|}^{2}=\sum _{i}{m}_{i}{p}_{i}$, where $\overline{M}$ is the average value of the observable $\hat{M}$, |*m*_*i*_) is the eigenstate of $\hat{M}$ with the eigenvalue *m*_*i*_, ${p}_{i}=|(\chi |{m}_{i}){|}^{2}$ is the probability of eigenvalue *m*_*i*_, |*χ*) is the input state. For the measurement of single observable, we only need to establish its eigenstates, and map the basis vectors of input cetrit state to the three eigenstates to obtain the probabilities of eigenvalues. For the measurement of two compatible observables $\overline{{A^{\prime} }_{i}{A^{\prime} }_{j}}$, we use joint measurement method^21,49^, which is that the input state projects onto the mutual eigenstate of the two compatible observables.

Corresponding to the joint measurement of two compatible observables, the establishment of the mutual eigenstate of the observables is a key task. As shown in the measurement stage of Fig. 2, we use the HWPs and PBSs to construct the desirable eigenstates. Taking the measurement of the compatible observables $\overline{{A^{\prime} }_{9}{A^{\prime} }_{10}}$ as an example, we appropriately arrange the experiment devices and set up the angles of HWP5 and HWP6, and the required mutual eigenstates can be established. With assuming that the input base vectors all are unit vectors, under the setting angles 22.5° for HWP5 and 0° for HWP6, the state vectors at output ports PD1, PD2 and PD3 are expressed as $\tfrac{\sqrt{2}}{2}|{\overrightarrow{{\rm{e}}}}_{0})+\tfrac{\sqrt{2}}{2}|{\overrightarrow{{\rm{e}}}}_{1})$, $-\,\tfrac{\sqrt{2}}{2}|{\overrightarrow{{\rm{e}}}}_{0})+\tfrac{\sqrt{2}}{2}|{\overrightarrow{{\rm{e}}}}_{1})$ and $|{\overrightarrow{{\rm{e}}}}_{2})$, respectively. Thus, the state vector at the output port PD1 is the eigenstate of ${A^{\prime} }_{9}=-\,1$ and ${A^{\prime} }_{10}=+\,1$; the state vector at the output port PD2 corresponds to the eigenstate of ${A^{\prime} }_{9}=+\,1$ and ${A^{\prime} }_{10}=-\,1$; the state vector at the output port PD3 describe the eigenstate of ${A^{\prime} }_{9}=+\,1$ and ${A^{\prime} }_{10}=+\,1$. Where ${A^{\prime} }_{9}=+\,1\,(\,-\,1)$ indicates that its eigenvalue is +1 (−1). These eigenstates meet the requirement of the above-mentioned joint measurement.

When the input state projects onto the eigenstates at the three output ports, namely the input base vectors are mapped to the polarization mode at the three output ports, we measure the optical intensities at these output ports. Then the optical intensities are normalized, namely, the optical intensity at each output port is divided by the total optical intensities, the probabilities of these eigenvalues can be obtained^47^. The probabilities at output port PD1, PD2 and PD3 are expressed as $P({A^{\prime} }_{9}=-\,1,{A^{\prime} }_{10}=+\,1)$, $P({A^{\prime} }_{9}=+\,1,\,{A^{\prime} }_{10}=-\,1)$ and $P({A^{\prime} }_{9}=+\,1,\,{A^{\prime} }_{10}=+\,1)$, respectively. Based on these probabilities, the average value of the correlation pair ${A^{\prime} }_{9}{A^{\prime} }_{10}$ can be calculated by $\overline{{A^{\prime} }_{9}{A^{\prime} }_{10}}=-\,P({A^{\prime} }_{9}=-\,1,{A^{\prime} }_{10}=+\,1)-P({A^{\prime} }_{9}=+\,1,{A^{\prime} }_{10}=-\,1)+P({A^{\prime} }_{9}\,=$ $+\,1,{A^{\prime} }_{10}=+\,1)$. Meanwhile, the average value of ${a^{\prime} }_{9}$ can also be obtained. At this moment, we do not need to consider ${a^{\prime} }_{10}$, and only ${A^{\prime} }_{9}$ is considered. The probabilities of ${A^{\prime} }_{9}$ at the output ports PD1, PD2 and PD3 are $P({A^{\prime} }_{9}=-\,1)$, $P({A^{\prime} }_{9}=+\,1)$ and $P({A^{\prime} }_{9}=+\,1)$, respectively. Thus, we can obtain $\overline{{A^{\prime} }_{9}}=-\,P({A^{\prime} }_{9}=-\,1)+P({A^{\prime} }_{9}=+\,1)+P({A^{\prime} }_{9}=+\,1)$. Here the optical intensities are detected by the photoelectric detectors (PDs).

For all other compatible observables ${A^{\prime} }_{i}{A^{\prime} }_{j}$, their mutual eigenstates can be obtained when the angles of the HWP5 and HWP6 are set up appropriately. Following the projection joint measurement, the probabilities $P({A^{\prime} }_{i}\,=-\,1,\,{A^{\prime} }_{j}=+\,1)$, $P({A^{\prime} }_{i}=+\,1,{A^{\prime} }_{j}=-\,1)$, and $P({A^{\prime} }_{i}=+\,1,{A^{\prime} }_{j}=+\,1)$ can also be obtained by measuring the optical intensities at the output ports. Thereupon the average values of ${A^{\prime} }_{i}{A^{\prime} }_{j}$ can be calculated by $\overline{{A^{\prime} }_{i}{A^{\prime} }_{j}}=-\,P({A^{\prime} }_{i}=-\,1,{A^{\prime} }_{j}=+\,1)-P({A^{\prime} }_{i}=+\,1,{A^{\prime} }_{j}=-\,1)+P({A^{\prime} }_{i}=+\,1,{A^{\prime} }_{j}=+\,1)$, and the average values of 13 single observables can be also obtained. The setting angles of HWP5 and HWP6 for all the observable measurements (13 observables and 24 compatible observable pairs) are listed in the table (see Methods section: The setting angles of HWPs for the observable measurements and the measurement methods for all observables).

In fact, the all 25 vectors (contain the dropped 12 vectors) and 48 orthogonalities shown in ref.^15^ are involved in the experiment. For simplicity, we only give an example ${a^{\prime} }_{9}$ and ${a^{\prime} }_{10}$ (the measurement for $\overline{{A^{\prime} }_{9}{A^{\prime} }_{10}}$), but all 16 triplets of mutually orthogonal vectors and all 25 vectors are given and are listed in the tables (see Methods section: The setting angles of HWPs for the observable measurements and the measurement methods for all observables). They can be obtained by appropriately setting the angles of HWP5 and HWP6. For instance, for the triplets (unnormalized) {(1, 1, −1), (2, −1, 1), (0, 1, 1)}, {(0, 1, −1), (1, 0, 0), (0, 1, 1)}, {(0, 1, −1), (2, 1, 1), (−1, 1, 1)}^15^, they correspond to (unnormalized) {${a^{\prime} }_{3}({h^{\prime} }_{3})=(1,1,-\,1)$, ${A^{\prime} }_{3hc}=(2,-\,1,1)$, ${a^{\prime} }_{5}({y}_{1}^{^{\prime} +})=(0,1,1)$}, {${a^{\prime} }_{6}({y}_{1}^{^{\prime} -})=(0,1,-\,1)$, ${a^{\prime} }_{11}({z^{\prime} }_{11})=(1,0,0)$, ${a^{\prime} }_{5}({y}_{1}^{^{\prime} +})=(0,\,1,\,1)$}, {${a^{\prime} }_{6}({y}_{1}^{^{\prime} -})=(0,1,-\,1)$, ${A^{\prime} }_{1hc}=(2,1,1)$, ${a^{\prime} }_{1}({h^{\prime} }_{1})=(-\,1,1,1)$}. For the additional 12 vectors, they act as the eigenstates of observables and observable pairs and also contribute to data used to form the statistics. In our experiment the optical intensity is the square of the vector product of the input state and the eigenstate^47^. Thus, after the projection measurements are implemented, the probabilities of eigenvalues are just the normalized optical intensities.

According to the above-mentioned measurement method, the experimental average values of 13 observables and 24 correlation pairs for seven different input states can be obtained. Therefore, the result of contextuality for the original Yu-Oh form in Eq. (4) can be calculated. The observable values for the input state $\tfrac{1}{\sqrt{3}}(|{\overrightarrow{{\rm{e}}}}_{0})+|{\overrightarrow{{\rm{e}}}}_{1})+|{\overrightarrow{{\rm{e}}}}_{2}))$ are listed in Table 2. The observable values for the six other input states are listed in the tables (see Methods section: The experimental values and theoretical results of observables for the different input states), and the experimental contextuality results for the seven input states are summarized in Table 3. After these results for the different input states are obtained, we can compare them with the noncontextual results and theoretical maximum predictions.

**Table 2:** Experimental average values and theoretical results of observables for the input state $\tfrac{1}{\sqrt{3}}(|{\overrightarrow{{\rm{e}}}}_{0})+|{\overrightarrow{{\rm{e}}}}_{1}\,)+|{\overrightarrow{{\rm{e}}}}_{2}))$, and the experimental contextuality results for the original Yu-Oh form. The dates in the parentheses behind the experimental averages are standard deviations.

Terms	Experimental value	Theoretical prediction	Terms	Experimental value	Theoretical prediction	Terms	Experimental value	Theoretical prediction
$\overline{{A^{\prime} }_{1}}$	0.777 (1)	0.778	$\overline{{A^{\prime} }_{1}{A^{\prime} }_{6}}$	0.704 (7)	0.778	$\overline{{A^{\prime} }_{5}{A^{\prime} }_{6}}$	−0.370 (5)	−0.333
$\overline{{A^{\prime} }_{2}}$	0.742 (2)	0.778	$\overline{{A^{\prime} }_{1}{A^{\prime} }_{7}}$	−0.524 (5)	−0.556	$\overline{{A^{\prime} }_{5}{A^{\prime} }_{11}}$	−0.842 (4)	−1
$\overline{{A^{\prime} }_{3}}$	0.770 (12)	0.778	$\overline{{A^{\prime} }_{1}{A^{\prime} }_{9}}$	−0.503 (1)	−0.556	$\overline{{A^{\prime} }_{6}{A^{\prime} }_{11}}$	0.212 (1)	0.333
$\overline{{A^{\prime} }_{4}}$	−0.892 (1)	−1	$\overline{{A^{\prime} }_{2}{A^{\prime} }_{5}}$	−0.529 (1)	−0.556	$\overline{{A^{\prime} }_{7}{A^{\prime} }_{8}}$	−0.337 (18)	−0.333
$\overline{{A^{\prime} }_{5}}$	−0.212 (1)	−0.333	$\overline{{A^{\prime} }_{2}{A^{\prime} }_{8}}$	0.703 (2)	0.778	$\overline{{A^{\prime} }_{7}{A^{\prime} }_{12}}$	−0.916 (6)	−1
$\overline{{A^{\prime} }_{6}}$	0.842 (4)	1	$\overline{{A^{\prime} }_{2}{A^{\prime} }_{9}}$	−0.599 (11)	−0.556	$\overline{{A^{\prime} }_{8}{A^{\prime} }_{12}}$	0.253 (12)	0.333
$\overline{{A^{\prime} }_{7}}$	−0.253 (12)	−0.333	$\overline{{A^{\prime} }_{3}{A^{\prime} }_{5}}$	−0.561 (21)	−0.556	$\overline{{A^{\prime} }_{9}{A^{\prime} }_{10}}$	−0.366 (14)	−0.333
$\overline{{A^{\prime} }_{8}}$	0.916 (6)	1	$\overline{{A^{\prime} }_{3}{A^{\prime} }_{7}}$	−0.543 (26)	−0.556	$\overline{{A^{\prime} }_{9}{A^{\prime} }_{13}}$	−0.920 (6)	−1
$\overline{{A^{\prime} }_{9}}$	−0.286 (11)	−0.333	$\overline{{A^{\prime} }_{3}{A^{\prime} }_{10}}$	0.702 (3)	0.778	$\overline{{A^{\prime} }_{10}{A^{\prime} }_{13}}$	0.286 (11)	0.333
$\overline{{A^{\prime} }_{10}}$	0.920 (6)	1	$\overline{{A^{\prime} }_{4}{A^{\prime} }_{6}}$	−0.919 (2)	−1	$\overline{{A^{\prime} }_{11}{A^{\prime} }_{12}}$	−0.308 (1)	−0.333
$\overline{{A^{\prime} }_{11}}$	0.342 (2)	0.333	$\overline{{A^{\prime} }_{4}{A^{\prime} }_{8}}$	−0.926 (1)	−1	$\overline{{A^{\prime} }_{11}{A^{\prime} }_{13}}$	−0.350 (2)	−0.333
$\overline{{A^{\prime} }_{12}}$	0.350 (2)	0.333	$\overline{{A^{\prime} }_{4}{A^{\prime} }_{10}}$	−0.935 (6)	−1	$\overline{{A^{\prime} }_{12}{A^{\prime} }_{13}}$	−0.342 (2)	−0.333
$\overline{{A^{\prime} }_{13}}$	0.308 (1)	0.333						
Original Yu-Oh	$\mathop{\sum }\limits_{i=1}^{13}\overline{{A^{\prime} }_{i}}-\frac{1}{4}\mathop{\sum }\limits_{i=1}^{13}\mathop{\sum }\limits_{j=1,j\ne i}^{13}{\Gamma }_{i,j}\overline{{A^{\prime} }_{i}{A^{\prime} }_{j}}=8.289\pm 0.073$

**Table 3:** Experimental results and the errors for the original form^14^ of the Yu-Oh inequality for the seven input states. The theoretical predictions for all input states are $\tfrac{25}{3}$, and the noncontextual results are 8.

Input states	Experimental value	Errors
$|{\overrightarrow{{\rm{e}}}}_{0})$	8.287	±0.089
$|{\overrightarrow{{\rm{e}}}}_{1})$	8.275	±0.072
$|{\overrightarrow{{\rm{e}}}}_{2})$	8.314	±0.086
$\tfrac{1}{\sqrt{2}}(|{\overrightarrow{{\rm{e}}}}_{0})+|{\overrightarrow{{\rm{e}}}}_{1}))$	8.246	±0.086
$\tfrac{1}{\sqrt{2}}(|{\overrightarrow{{\rm{e}}}}_{0})+|{\overrightarrow{{\rm{e}}}}_{2}))$	8.189	±0.068
$\tfrac{1}{\sqrt{2}}(|{\overrightarrow{{\rm{e}}}}_{1})+|{\overrightarrow{{\rm{e}}}}_{2}))$	8.205	±0.082
$\tfrac{1}{\sqrt{3}}(|{\overrightarrow{{\rm{e}}}}_{0})+|{\overrightarrow{{\rm{e}}}}_{1})+|{\overrightarrow{{\rm{e}}}}_{2}))$	8.289	±0.073

It can be seen from Table 3 that the original Yu-Oh inequality shows the violations for seven different input states. The experimental average result for seven different input states is $8.258\pm 0.079$, which exceeds the noncontextual bound 8 by 3.2%, and the results show the favorable state-independent contextuality phenomenon. But they have some deviations from the theoretical maximum prediction $\tfrac{25}{3}$. These are caused by the experimental imperfections, such as the imperfect PBS and HWP. Despite existence of these imperfections, the results in classical optical experiment still show the large violations of the noncontextual inequality, which are agreement with the theoretical prediction.

### Experimental demonstration of state-independent contextuality for the improved and optimal forms of Yu-Oh inequality in classical light systems

In this section, the experimental demonstration of state-independent contextuality for the improved and optimal forms of Yu-Oh inequality is given in classical light. Based on the experimental setup in Fig. 2, the violations of the improved and optimal forms of Yu-Oh inequality can also be testified in classical optical systems. Similarly, the experiment processes require the input states preparations and observable measurements. We can prepare the different input states by using the method in the state preparation stage. For the measurements of the single observable and compatible observable pairs, the methods are also the same to the measurements of observables in original Yu-Oh form, namely, establishing the eigenstates, implementing the projection measurements, measuring optical intensities to obtain the probabilities of eigenvalues, and calculating the average values of observables. However, in inequality Eq. (7) three compatible observables $${A^{\prime} }_{i}{A^{\prime} }_{j}{A^{\prime} }_{k}$$ are involved. Their mutual eigenstates can be also established, thus the joint probabilities $$P({A^{\prime} }_{i}\,=-\,1,{A^{\prime} }_{j}=+\,1,{A^{\prime} }_{k}=+\,1)$$, $$P({A^{\prime} }_{i}=+\,1,{A^{\prime} }_{j}=-\,1,{A^{\prime} }_{k}=+\,1)$$ and $$P({A^{\prime} }_{i}=+\,1,{A^{\prime} }_{j}=+\,1,{A^{\prime} }_{k}=-\,1)$$ can be measured at the three output ports. Similarly, the average value of $${A^{\prime} }_{i}{A^{\prime} }_{j}{A^{\prime} }_{k}$$ can be calculated by $$\overline{{A^{\prime} }_{i}{A^{\prime} }_{j}{A^{\prime} }_{k}}=-\,\,P({A^{\prime} }_{i}=-\,1,{A^{\prime} }_{j}=+\,1,$$ $${A^{\prime} }_{k}=+\,1)$$ − $$P({A^{\prime} }_{i}=+\,1,{A^{\prime} }_{j}=-\,1,{A^{\prime} }_{k}\,=+\,1)$$ $$-P({A^{\prime} }_{i}=+\,1,{A^{\prime} }_{j}=$$ $$+\,1,{A^{\prime} }_{k}=-\,1)$$. All observables are measured, thus the contextuality results in Eqs (5–7) can be obtained. For example, using the equation $$\mathop{\sum }\limits_{i=1}^{6}\overline{{A^{\prime} }_{i}}+2\mathop{\sum }\limits_{j=7}^{11}\overline{{A^{\prime} }_{j}}+3\mathop{\sum }\limits_{k=12}^{13}\overline{{A^{\prime} }_{k}}\,-\mathop{\sum }\limits_{i=1}^{4}\mathop{\sum }\limits_{j=5}^{10}{\Gamma }_{i,j}\overline{{A^{\prime} }_{i}{A^{\prime} }_{j}}$$ $$-\,\mathop{\sum }\limits_{m=5}^{6}\overline{{A^{\prime} }_{m}{A^{\prime} }_{11}}\,-\,2\mathop{\sum }\limits_{m=7}^{10}\mathop{\sum }\limits_{n > m}^{13}{\Gamma }_{m,n}\overline{{A^{\prime} }_{m}{A^{\prime} }_{n}}-\,\mathop{\sum }\limits_{n=12}^{13}$$$$\overline{{A^{\prime} }_{11}{A^{\prime} }_{n}}-2\overline{{A^{\prime} }_{12}{A^{\prime} }_{13}}$$ and measured data shown in Table 2, we obtain the contextuality result $$17.181\pm 0.087$$ corresponding to the input state $$\tfrac{1}{\sqrt{3}}(|{\overrightarrow{{\rm{e}}}}_{0})+|{\overrightarrow{{\rm{e}}}}_{1}\,)+|{\overrightarrow{{\rm{e}}}}_{2}))$$ for the optimal form opt_2_. Of course, for the six other input states, we can also obtain the contextuality values by using the data shown in the tables (see Methods section: The experimental values and theoretical results of observables for the different input states).

Following the similar method, the contextuality results of the improved form, and the optimal forms opt_2_ and opt_3_ for the different input states can also be obtained. For these modified contextuality forms, the concrete calculations are not given, and here we only list the final experimental results. For the improved form of Yu-Oh inequality in ref.^16^, the experimental results of contextuality are showed in Table 4. The experimental average result for the seven input states is $9.622\pm 0.067$, which exceeds the noncontextual bounds 9 by 6.9%. The large noncontextual violations are revealed. For the optimal forms opt_2_ and opt_3_ in ref.^17^, the experimental results of contextuality are showed in Tables 5 and 6, respectively. The average values of the experiment results for opt_2_ and opt_3_ are $17.244\pm 0.088$ and $27.505\pm 0.099$, respectively, and they exceed the noncontextual bounds 16 by 7.8% and 25 by 10.0%. These results show the clear-cut noncontextual violations. This means that the state-independent contextualities for the different Yu-Oh forms are testified in classical optical systems. In addition, the violation degrees for the optimal forms are larger than those for the original form and the improved form, which shows that the optimal forms are tighter.

**Table 4:** Experimental results and the errors for the improved form^16^ of the Yu-Oh inequality for the seven input states. The theoretical predictions for all input states are $\tfrac{29}{3}$, and the noncontextual results are 9.

Input states	Experimental value	Errors
$|{\overrightarrow{{\rm{e}}}}_{0})$	9.630	±0.087
$|{\overrightarrow{{\rm{e}}}}_{1})$	9.622	±0.057
$|{\overrightarrow{{\rm{e}}}}_{2})$	9.653	±0.068
$\tfrac{1}{\sqrt{2}}(|{\overrightarrow{{\rm{e}}}}_{0})+|{\overrightarrow{{\rm{e}}}}_{1}))$	9.660	±0.072
$\tfrac{1}{\sqrt{2}}(|{\overrightarrow{{\rm{e}}}}_{0})+|{\overrightarrow{{\rm{e}}}}_{2}))$	9.580	±0.055
$\tfrac{1}{\sqrt{2}}(|{\overrightarrow{{\rm{e}}}}_{1})+|{\overrightarrow{{\rm{e}}}}_{2}))$	9.619	±0.064
$\tfrac{1}{\sqrt{3}}(|{\overrightarrow{{\rm{e}}}}_{0})+|{\overrightarrow{{\rm{e}}}}_{1})+|{\overrightarrow{{\rm{e}}}}_{2}))$	9.591	±0.063

**Table 5:** Experimental results and the errors for the optimal form opt_2_^17^ of the Yu-Oh inequality for the seven input states. The theoretical predictions for all input states are $\tfrac{52}{3}$, and the noncontextual results are 16.

Input states	Experimental value	Errors
$|{\overrightarrow{{\rm{e}}}}_{0})$	17.260	±0.106
$|{\overrightarrow{{\rm{e}}}}_{1})$	17.245	±0.078
$|{\overrightarrow{{\rm{e}}}}_{2})$	17.305	±0.093
$\tfrac{1}{\sqrt{2}}(|{\overrightarrow{{\rm{e}}}}_{0})+|{\overrightarrow{{\rm{e}}}}_{1}))$	17.320	±0.090
$\tfrac{1}{\sqrt{2}}(|{\overrightarrow{{\rm{e}}}}_{0})+|{\overrightarrow{{\rm{e}}}}_{2}))$	17.161	±0.071
$\tfrac{1}{\sqrt{2}}(|{\overrightarrow{{\rm{e}}}}_{1})+|{\overrightarrow{{\rm{e}}}}_{2}))$	17.238	±0.094
$\tfrac{1}{\sqrt{3}}(|{\overrightarrow{{\rm{e}}}}_{0})+|{\overrightarrow{{\rm{e}}}}_{1})+|{\overrightarrow{{\rm{e}}}}_{2}))$	17.181	±0.087

**Table 6:** Experimental results and the errors for the optimal form opt_3_^17^ of the Yu-Oh inequality for the seven input states. The theoretical predictions for all input states are $\tfrac{83}{3}$, and the noncontextual results are 25.

Input states	Experimental value	Errors
$|{\overrightarrow{{\rm{e}}}}_{0})$	27.523	±0.139
$|{\overrightarrow{{\rm{e}}}}_{1})$	27.489	±0.081
$|{\overrightarrow{{\rm{e}}}}_{2})$	27.510	±0.105
$\tfrac{1}{\sqrt{2}}(|{\overrightarrow{{\rm{e}}}}_{0})+|{\overrightarrow{{\rm{e}}}}_{1}))$	27.518	±0.100
$\tfrac{1}{\sqrt{2}}(|{\overrightarrow{{\rm{e}}}}_{0})+|{\overrightarrow{{\rm{e}}}}_{2}))$	27.349	±0.087
$\tfrac{1}{\sqrt{2}}(|{\overrightarrow{{\rm{e}}}}_{1})+|{\overrightarrow{{\rm{e}}}}_{2}))$	27.564	±0.088
$\tfrac{1}{\sqrt{3}}(|{\overrightarrow{{\rm{e}}}}_{0})+|{\overrightarrow{{\rm{e}}}}_{1})+|{\overrightarrow{{\rm{e}}}}_{2}))$	27.582	±0.091

## Conclusions

In summary, we have simulated experimentally the state-independent contextuality in the classical optical systems. Based on the path and polarization degrees of freedom of classical optics fields, we have constructed the cetrit. Furthermore, the different input states have been established. Using projective measurement, the average values of the observables and observable correlation pairs have been obtained, and the results of contextuality for different Yu-Oh forms have been calculated. The violation of the original form of Yu-Oh inequality has not only been observed, the violations of the improved and optimal forms of Yu-Oh inequality for different input states have also been observed in the classical optical experiments. In our opinion, Maxwell’s equations in the paraxial ray approximation describing classical optical field have a form similar to the Schrodinger equation describing quantum mechanics, and such a correspondence leads to the analogy between quantum mechanics and classical optics to be made. Thus, our results not only provide new physical insights into the contextuality, but also enrich the theory of classical optical coherence and show the application prospect of the concepts developed recently in quantum information science to classical optical systems and optical information processes.

## Methods

### The setting angles of HWPs for the different input state preparations

In order to test the inequality (4), namely the original Yu-Oh inequality, the operations about the state preparations and the observable measurements are implemented. The setting angles of HWPs and some details for the observable measurements are given as follow. Of course, these operations for the different forms of Yu-Oh inequality are the same basically. In the state preparation stage, the setting angles of HWP1 and HWP2 for the seven different input states are summarized in Table 7.

**Table 7:** The setting angles of HWPs for the preparations of seven different input states.

Input state	HWP1 *θ*_1_	HWP2 *θ*_2_
$|{\overrightarrow{{\rm{e}}}}_{0})$	0°	0°
$|{\overrightarrow{{\rm{e}}}}_{1})$	−45°	0°
$|{\overrightarrow{{\rm{e}}}}_{2})$	45°	45°
$\tfrac{1}{\sqrt{2}}(|{\overrightarrow{{\rm{e}}}}_{0})+|{\overrightarrow{{\rm{e}}}}_{1}))$	22.5°	90°
$\tfrac{1}{\sqrt{2}}(|{\overrightarrow{{\rm{e}}}}_{0})+|{\overrightarrow{{\rm{e}}}}_{2}))$	22.5°	45°
$\tfrac{1}{\sqrt{2}}(|{\overrightarrow{{\rm{e}}}}_{1})+|{\overrightarrow{{\rm{e}}}}_{2}))$	−45°	−22.5°
$\tfrac{1}{\sqrt{3}}(|{\overrightarrow{{\rm{e}}}}_{0})+|{\overrightarrow{{\rm{e}}}}_{1})+|{\overrightarrow{{\rm{e}}}}_{2}))$	−27.37°	−22.5°

### The setting angles of HWPs for the observable measurements and the measurement methods for all observables

In order to test these observables ${A^{\prime} }_{i}$ and observable pairs ${A^{\prime} }_{i}{A^{\prime} }_{j}$, the angles of HWP5 and HWP6 need to be set up appropriately. In Table 8, the setting angles of HWP5 and HWP6 for the observable measurements are listed.

**Table 8:** The setting angles of HWP5 and 6 for the observable measurements. The observables (vectors) in parentheses are the observables in Yu-Oh’s scenario, and the vectors in square brackets are the additional vectors in ref.^15^.

Observables	PD1	PD2	PD3	HWP5	HWP6
${A^{\prime} }_{1}{A^{\prime} }_{9}$	(${a^{\prime} }_{9}$)	(${a^{\prime} }_{1}$)	[${A^{\prime} }_{1h}$]	22.5°	17.6°
${A^{\prime} }_{2}{A^{\prime} }_{9}$	(${a^{\prime} }_{9}$)	(${a^{\prime} }_{2}$)	[${A^{\prime} }_{2h}$]	22.5°	−17.6°
${A^{\prime} }_{3}{A^{\prime} }_{10}$	(${a^{\prime} }_{10}$)	(${a^{\prime} }_{3}$)	[${A^{\prime} }_{3h}$]	67.5°	17.6°
${A^{\prime} }_{4}{A^{\prime} }_{10}$	(${a^{\prime} }_{10}$)	(${a^{\prime} }_{4}$)	[${A^{\prime} }_{4h}$]	−22.5°	17.6°
${A^{\prime} }_{5}{A^{\prime} }_{6}$, ${A^{\prime} }_{5}{A^{\prime} }_{11}$, ${A^{\prime} }_{6}{A^{\prime} }_{11}$	(${a^{\prime} }_{11}$)	(${a^{\prime} }_{5}$)	(${a^{\prime} }_{6}$)	0°	22.5°
${A^{\prime} }_{7}{A^{\prime} }_{8}$, ${A^{\prime} }_{7}{A^{\prime} }_{12}$, ${A^{\prime} }_{8}{A^{\prime} }_{12}$	(${a^{\prime} }_{12}$)	(${a^{\prime} }_{8}$)	(${a^{\prime} }_{7}$)	45°	22.5°
${A^{\prime} }_{9}{A^{\prime} }_{10}$, ${A^{\prime} }_{9}{A^{\prime} }_{13}$, ${A^{\prime} }_{10}{A^{\prime} }_{13}$	(${a^{\prime} }_{9}$)	(${a^{\prime} }_{10}$)	(${a^{\prime} }_{13}$)	22.5°	0°
${A^{\prime} }_{11}{A^{\prime} }_{12}$, ${A^{\prime} }_{11}{A^{\prime} }_{13}$, ${A^{\prime} }_{12}{A^{\prime} }_{13}$	(${a^{\prime} }_{11}$)	(${a^{\prime} }_{12}$)	(${a^{\prime} }_{13}$)	0°	0°

As shown in Table 8, there are extra state vectors ${A^{\prime} }_{1h}$, ${A^{\prime} }_{2h}$, ${A^{\prime} }_{3h}$ and ${A^{\prime} }_{4h}$ at the output port PD3. They are the eigenstates with the eigenvalue +1 of the two observables, whose corresponding vectors are located at the output ports PD1 and PD2, and these extra state vectors are expressed as ${A^{\prime} }_{1h}\,=\tfrac{\sqrt{6}}{6}|{\overrightarrow{{\rm{e}}}}_{0})-\tfrac{\sqrt{6}}{6}|{\overrightarrow{{\rm{e}}}}_{1})+\tfrac{\sqrt{6}}{3}|{\overrightarrow{{\rm{e}}}}_{2})$, ${A^{\prime} }_{2h}\,=-\,\tfrac{\sqrt{6}}{6}|{\overrightarrow{{\rm{e}}}}_{0})\,+\,\tfrac{\sqrt{6}}{6}|{\overrightarrow{{\rm{e}}}}_{1})\,+\,\tfrac{\sqrt{6}}{3}|{\overrightarrow{{\rm{e}}}}_{2})$, ${A^{\prime} }_{3h}\,=\tfrac{\sqrt{6}}{6}|{\overrightarrow{{\rm{e}}}}_{0})\,+\,\tfrac{\sqrt{6}}{6}|{\overrightarrow{{\rm{e}}}}_{1})\,+\,\tfrac{\sqrt{6}}{3}|{\overrightarrow{{\rm{e}}}}_{2})$ and ${A^{\prime} }_{4h}\,=-\,\tfrac{\sqrt{6}}{6}|{\overrightarrow{{\rm{e}}}}_{0})-\tfrac{\sqrt{6}}{6}|{\overrightarrow{{\rm{e}}}}_{1})+$ $\tfrac{\sqrt{6}}{3}|{\overrightarrow{{\rm{e}}}}_{2})$. We can see that all 13 observables are contained in Table 8, but the 24 correlation pairs are not contained entirely. For the rest of correlation pairs, the additional experimental designs are needed. It is implemented by changing the first four columns in Table 8. The concrete methods are that the basis vectors are exchanged. Following this operation, $|{\overrightarrow{{\rm{e}}}}_{0})$ and $|{\overrightarrow{{\rm{e}}}}_{2})$ are exchanged, and a parts of correlation pairs can be obtained. Exchanging the basis vectors $|{\overrightarrow{{\rm{e}}}}_{1})$ and $|{\overrightarrow{{\rm{e}}}}_{2})$, the other parts of correlation pairs can also be obtained. As a result, all correlation pairs (24 pairs) can be gotten, and these exchange processes are showed in Table 9.

**Table 9:** The exchanges of the input basis vectors for the measurement of the other correlation pairs. The meanings of parentheses and square brackets are same as Table 8.

	Observables	PD1	PD2	PD3	HWP5	HWP6
$|{\overrightarrow{{\rm{e}}}}_{0})\,\leftrightarrow \,|{\overrightarrow{{\rm{e}}}}_{2})$	${A^{\prime} }_{3}{A^{\prime} }_{5}$	(${a^{\prime} }_{5}$)	(${a^{\prime} }_{3}$)	[${A^{\prime} }_{3hc}$]	22.5°	17.6°
	${A^{\prime} }_{2}{A^{\prime} }_{5}$	(${a^{\prime} }_{5}$)	(${a^{\prime} }_{2}$)	[${A^{\prime} }_{2hc}$]	22.5°	−17.6°
	${A^{\prime} }_{1}{A^{\prime} }_{6}$	(${a^{\prime} }_{6}$)	(${a^{\prime} }_{1}$)	[${A^{\prime} }_{1hc}$]	67.5°	17.6°
	${A^{\prime} }_{4}{A^{\prime} }_{6}$	(${a^{\prime} }_{6}$)	(${a^{\prime} }_{4}$)	[${A^{\prime} }_{4hc}$]	−22.5°	17.6°
$|{\overrightarrow{{\rm{e}}}}_{1})\,\leftrightarrow \,|{\overrightarrow{{\rm{e}}}}_{2})$	${A^{\prime} }_{1}{A^{\prime} }_{7}$	(${a^{\prime} }_{7}$)	(${a^{\prime} }_{1}$)	[${A^{\prime} }_{1hd}$]	22.5°	17.6°
	${A^{\prime} }_{3}{A^{\prime} }_{7}$	(${a^{\prime} }_{7}$)	(${a^{\prime} }_{3}$)	[${A^{\prime} }_{3hd}$]	22.5°	−17.6°
	${A^{\prime} }_{2}{A^{\prime} }_{8}$	(${a^{\prime} }_{8}$)	(${a^{\prime} }_{2}$)	[${A^{\prime} }_{2hd}$]	67.5°	17.6°
	${A^{\prime} }_{4}{A^{\prime} }_{8}$	(${a^{\prime} }_{8}$)	(${a^{\prime} }_{4}$)	[${A^{\prime} }_{4hd}$]	−22.5°	17.6°

Because of the exchanges of the basis vectors for the input states, the sate vectors at the output port PD3 are also changed. For the exchange $|{\overrightarrow{{\rm{e}}}}_{0})\leftrightarrow |{\overrightarrow{{\rm{e}}}}_{2})$, the exchange of the basis vectors $|{\overrightarrow{{\rm{e}}}}_{0})$ and $|{\overrightarrow{{\rm{e}}}}_{2})$ of ${A^{\prime} }_{1h}$, ${A^{\prime} }_{2h}$, ${A^{\prime} }_{3h}$ and ${A^{\prime} }_{4h}$ in Table 8 is only needed, and the corresponding state vectors ${A^{\prime} }_{3hc}({A^{\prime} }_{3hc}=\tfrac{\sqrt{6}}{3}|{\overrightarrow{{\rm{e}}}}_{0})-\tfrac{\sqrt{6}}{6}|{\overrightarrow{{\rm{e}}}}_{1})+\tfrac{\sqrt{6}}{6}|{\overrightarrow{{\rm{e}}}}_{2}))$, ${A^{\prime} }_{2hc}({A^{\prime} }_{2hc}=\tfrac{\sqrt{6}}{3}|{\overrightarrow{{\rm{e}}}}_{0})+\tfrac{\sqrt{6}}{6}|{\overrightarrow{{\rm{e}}}}_{1})-\tfrac{\sqrt{6}}{6}|{\overrightarrow{{\rm{e}}}}_{2}))$, ${A^{\prime} }_{1hc}({A^{\prime} }_{1hc}=\tfrac{\sqrt{6}}{3}|{\overrightarrow{{\rm{e}}}}_{0})+\tfrac{\sqrt{6}}{6}|{\overrightarrow{{\rm{e}}}}_{1})+\tfrac{\sqrt{6}}{6}|{\overrightarrow{{\rm{e}}}}_{2}))$ and ${A^{\prime} }_{4hc}({A^{\prime} }_{4hc}=$ $\tfrac{\sqrt{6}}{3}|{\overrightarrow{{\rm{e}}}}_{0})-\tfrac{\sqrt{6}}{6}|{\overrightarrow{{\rm{e}}}}_{1})-\tfrac{\sqrt{6}}{6}|{\overrightarrow{{\rm{e}}}}_{2}))$ at the output port PD3 can be obtained. They are shown in Table 9. For the exchange $|{\overrightarrow{{\rm{e}}}}_{1})\leftrightarrow |{\overrightarrow{{\rm{e}}}}_{2})$, the exchange of the basis vectors $|{\overrightarrow{{\rm{e}}}}_{1})$ and $|{\overrightarrow{{\rm{e}}}}_{2})$ of ${A^{\prime} }_{1h}$, ${A^{\prime} }_{2h}$, ${A^{\prime} }_{3h}$ and ${A^{\prime} }_{4h}$ is only needed, the corresponding state vectors ${A^{\prime} }_{1hd}({A^{\prime} }_{1h{\rm{d}}}=\tfrac{\sqrt{6}}{6}|{\overrightarrow{{\rm{e}}}}_{0})+\tfrac{\sqrt{6}}{3}|{\overrightarrow{{\rm{e}}}}_{1})-\tfrac{\sqrt{6}}{6}|{\overrightarrow{{\rm{e}}}}_{2}))$, ${A^{\prime} }_{3hd}({A^{\prime} }_{3h{\rm{d}}}=-\,\tfrac{\sqrt{6}}{6}|{\overrightarrow{{\rm{e}}}}_{0})+\tfrac{\sqrt{6}}{3}|{\overrightarrow{{\rm{e}}}}_{1})+\tfrac{\sqrt{6}}{6}|{\overrightarrow{{\rm{e}}}}_{2}))$, ${A^{\prime} }_{2hd}({A^{\prime} }_{2h{\rm{d}}}=\tfrac{\sqrt{6}}{6}|{\overrightarrow{{\rm{e}}}}_{0})+\tfrac{\sqrt{6}}{3}|{\overrightarrow{{\rm{e}}}}_{1})+\tfrac{\sqrt{6}}{6}|{\overrightarrow{{\rm{e}}}}_{2}))$ and ${A^{\prime} }_{4hd}({A^{\prime} }_{4h{\rm{d}}}=-\,\tfrac{\sqrt{6}}{6}|{\overrightarrow{{\rm{e}}}}_{0})+\tfrac{\sqrt{6}}{3}|{\overrightarrow{{\rm{e}}}}_{1})-\tfrac{\sqrt{6}}{6}|{\overrightarrow{{\rm{e}}}}_{2}))$ can be obtained, and they are also shown in Table 9.

In fact, in Yu-Oh’s scenario, 13 vectors and 24 correlation pairs (orthogonal vectors) are involved, and the contextuality can be showed through these observables (vectors). Recently, Pavičić pointed out that there should be additional 12 vectors and all 48 orthogonalities in Yu-Oh’s sets^15^, and these are also exhibited in our experiment. In Tables 8 and 9, the vectors (observables) in each row correspond to the vectors on each edge in Fig. 19 of ref.^15^, and the 16 rows correspond to the all 16 edges. Thus, all 16 triplets of mutually orthogonal vectors, 25 vectors and 48 orthogonalities are contained. The vectors (observables) in parentheses are the vectors in the Yu-Oh’s scenario^14^, and there are 13 vectors (observables), which can compose the 24 correlation pairs. The vectors (observables) in square brackets are the dropped 12 vectors, which are illuminated in ref.^15^. For example, ${A^{\prime} }_{3hc}=\tfrac{\sqrt{6}}{3}|{\overrightarrow{{\rm{e}}}}_{0})-\tfrac{\sqrt{6}}{6}|{\overrightarrow{{\rm{e}}}}_{1})+\tfrac{\sqrt{6}}{6}|{\overrightarrow{{\rm{e}}}}_{2})$, ${a^{\prime} }_{3}$ (vector $\tfrac{\sqrt{3}}{3}|{\overrightarrow{{\rm{e}}}}_{0})+\tfrac{\sqrt{3}}{3}|{\overrightarrow{{\rm{e}}}}_{1})-\tfrac{\sqrt{3}}{3}|{\overrightarrow{{\rm{e}}}}_{2})$) and ${a^{\prime} }_{5}$ (vector $\tfrac{\sqrt{2}}{2}|{\overrightarrow{{\rm{e}}}}_{1})+\tfrac{\sqrt{2}}{2}|{\overrightarrow{{\rm{e}}}}_{2})$) compose the triplets of mutually orthogonal vectors, and they correspond to the triplets (unnormalized) {(2, −1, 1), (1, 1, −1), (0, 1, 1)} in Fig. 9 of ref.^15^. Our work is consistent with the description in ref.^15^.

### The experimental values and theoretical results of observables for the different input states

For the original Yu-Oh form, the experiment results and the theory predict values of the observables for six other input states are listed in Tables 10–15 as follow. The experimental results of contextuality are listed in the last row.

**Table 10:** Experimental average values and theoretical results of observables for the input state $|{\overrightarrow{{\rm{e}}}}_{0})$. The dates behind the experimental average values in the parentheses are standard deviations.

Terms	Experimental value	Theoretical prediction	Terms	Experimental value	Theoretical prediction	Terms	Experimental value	Theoretical prediction
$\overline{{A^{\prime} }_{1}}$	0.335 (24)	0.333	$\overline{{A^{\prime} }_{1}{A^{\prime} }_{6}}$	0.355 (8)	0.333	$\overline{{A^{\prime} }_{5}{A^{\prime} }_{6}}$	0.960 (2)	1
$\overline{{A^{\prime} }_{2}}$	0.316 (12)	0.333	$\overline{{A^{\prime} }_{1}{A^{\prime} }_{7}}$	−0.696 (7)	−0.667	$\overline{{A^{\prime} }_{5}{A^{\prime} }_{11}}$	−0.991 (1)	−1
$\overline{{A^{\prime} }_{3}}$	0.353 (15)	0.333	$\overline{{A^{\prime} }_{1}{A^{\prime} }_{9}}$	−0.665 (27)	−0.667	$\overline{{A^{\prime} }_{6}{A^{\prime} }_{11}}$	−0.969 (1)	−1
$\overline{{A^{\prime} }_{4}}$	0.311 (18)	0.333	$\overline{{A^{\prime} }_{2}{A^{\prime} }_{5}}$	0.364 (14)	0.333	$\overline{{A^{\prime} }_{7}{A^{\prime} }_{8}}$	−0.979 (0)	−1
$\overline{{A^{\prime} }_{5}}$	0.969 (1)	1	$\overline{{A^{\prime} }_{2}{A^{\prime} }_{8}}$	−0.683 (15)	−0.667	$\overline{{A^{\prime} }_{7}{A^{\prime} }_{12}}$	−0.009 (6)	0
$\overline{{A^{\prime} }_{6}}$	0.991 (1)	1	$\overline{{A^{\prime} }_{2}{A^{\prime} }_{9}}$	−0.652 (25)	−0.667	$\overline{{A^{\prime} }_{8}{A^{\prime} }_{12}}$	−0.013 (7)	0
$\overline{{A^{\prime} }_{7}}$	0.013 (7)	0	$\overline{{A^{\prime} }_{3}{A^{\prime} }_{5}}$	0.363 (6)	0.333	$\overline{{A^{\prime} }_{9}{A^{\prime} }_{10}}$	−0.982 (1)	−1
$\overline{{A^{\prime} }_{8}}$	0.009 (6)	0	$\overline{{A^{\prime} }_{3}{A^{\prime} }_{7}}$	−0.635 (16)	−0.667	$\overline{{A^{\prime} }_{9}{A^{\prime} }_{13}}$	−0.033 (1)	0
$\overline{{A^{\prime} }_{9}}$	−0.015 (2)	0	$\overline{{A^{\prime} }_{3}{A^{\prime} }_{10}}$	−0.686 (8)	−0.667	$\overline{{A^{\prime} }_{10}{A^{\prime} }_{13}}$	0.015 (2)	0
$\overline{{A^{\prime} }_{10}}$	0.033 (1)	0	$\overline{{A^{\prime} }_{4}{A^{\prime} }_{6}}$	0.353 (5)	0.333	$\overline{{A^{\prime} }_{11}{A^{\prime} }_{12}}$	−0.991 (1)	−1
$\overline{{A^{\prime} }_{11}}$	−0.973 (1)	−1	$\overline{{A^{\prime} }_{4}{A^{\prime} }_{8}}$	−0.684 (4)	−0.667	$\overline{{A^{\prime} }_{11}{A^{\prime} }_{13}}$	−0.982 (1)	−1
$\overline{{A^{\prime} }_{12}}$	0.982 (1)	1	$\overline{{A^{\prime} }_{4}{A^{\prime} }_{10}}$	−0.683 (22)	−0.667	$\overline{{A^{\prime} }_{12}{A^{\prime} }_{13}}$	0.973 (1)	1
$\overline{{A^{\prime} }_{13}}$	0.991 (1)	1						
Original Yu-Oh	$\mathop{\sum }\limits_{i=1}^{13}\overline{{A^{\prime} }_{i}}-\frac{1}{4}\mathop{\sum }\limits_{i=1}^{13}\mathop{\sum }\limits_{j=1,j\ne i}^{13}{\Gamma }_{i,j}\overline{{A^{\prime} }_{i}{A^{\prime} }_{j}}=8.287\pm 89$

**Table 11:** Experimental average values and theoretical results of observables for the input state $|{\overrightarrow{{\rm{e}}}}_{1})$. The dates behind the experimental average values in the parentheses are standard deviations.

Terms	Experimental value	Theoretical prediction	Terms	Experimental value	Theoretical prediction	Terms	Experimental value	Theoretical prediction
$\overline{{A^{\prime} }_{1}}$	0.322 (8)	0.333	$\overline{{A^{\prime} }_{1}{A^{\prime} }_{6}}$	−0.644 (1)	−0.667	$\overline{{A^{\prime} }_{5}{A^{\prime} }_{6}}$	−0.986 (4)	−1
$\overline{{A^{\prime} }_{2}}$	0.327 (3)	0.333	$\overline{{A^{\prime} }_{1}{A^{\prime} }_{7}}$	0.314 (8)	0.333	$\overline{{A^{\prime} }_{5}{A^{\prime} }_{11}}$	−0.021 (21)	0
$\overline{{A^{\prime} }_{3}}$	0.326 (4)	0.333	$\overline{{A^{\prime} }_{1}{A^{\prime} }_{9}}$	−0.668 (9)	−0.667	$\overline{{A^{\prime} }_{6}{A^{\prime} }_{11}}$	0.007 (22)	0
$\overline{{A^{\prime} }_{4}}$	0.330 (2)	0.333	$\overline{{A^{\prime} }_{2}{A^{\prime} }_{5}}$	−0.647 (3)	−0.667	$\overline{{A^{\prime} }_{7}{A^{\prime} }_{8}}$	0.963 (1)	1
$\overline{{A^{\prime} }_{5}}$	−0.007 (22)	0	$\overline{{A^{\prime} }_{2}{A^{\prime} }_{8}}$	0.320 (2)	0.333	$\overline{{A^{\prime} }_{7}{A^{\prime} }_{12}}$	−0.975 (0)	−1
$\overline{{A^{\prime} }_{6}}$	0.021 (21)	0	$\overline{{A^{\prime} }_{2}{A^{\prime} }_{9}}$	−0.662 (15)	−0.667	$\overline{{A^{\prime} }_{8}{A^{\prime} }_{12}}$	−0.988 (1)	−1
$\overline{{A^{\prime} }_{7}}$	0.988 (1)	1	$\overline{{A^{\prime} }_{3}{A^{\prime} }_{5}}$	−0.651 (5)	−0.667	$\overline{{A^{\prime} }_{9}{A^{\prime} }_{10}}$	−0.977 (2)	−1
$\overline{{A^{\prime} }_{8}}$	0.975 (0)	1	$\overline{{A^{\prime} }_{3}{A^{\prime} }_{7}}$	0.319 (3)	0.333	$\overline{{A^{\prime} }_{9}{A^{\prime} }_{13}}$	−0.006 (7)	0
$\overline{{A^{\prime} }_{9}}$	−0.017 (8)	0	$\overline{{A^{\prime} }_{3}{A^{\prime} }_{10}}$	−0.640 (12)	−0.667	$\overline{{A^{\prime} }_{10}{A^{\prime} }_{13}}$	−0.017 (8)	0
$\overline{{A^{\prime} }_{10}}$	0.006 (7)	0	$\overline{{A^{\prime} }_{4}{A^{\prime} }_{6}}$	−0.654 (5)	−0.667	$\overline{{A^{\prime} }_{11}{A^{\prime} }_{12}}$	−0.980 (1)	−1
$\overline{{A^{\prime} }_{11}}$	0.989 (1)	1	$\overline{{A^{\prime} }_{4}{A^{\prime} }_{8}}$	0.322 (2)	0.333	$\overline{{A^{\prime} }_{11}{A^{\prime} }_{13}}$	0.969 (1)	1
$\overline{{A^{\prime} }_{12}}$	−0.969 (1)	−1	$\overline{{A^{\prime} }_{4}{A^{\prime} }_{10}}$	−0.648 (4)	−0.667	$\overline{{A^{\prime} }_{12}{A^{\prime} }_{13}}$	−0.989 (1)	−1
$\overline{{A^{\prime} }_{13}}$	0.980 (1)	1						
Original Yu-Oh	$\mathop{\sum }\limits_{i=1}^{13}\overline{{A^{\prime} }_{i}}-\frac{1}{4}\mathop{\sum }\limits_{i=1}^{13}\mathop{\sum }\limits_{j=1,j\ne i}^{13}{\Gamma }_{i,j}\overline{{A^{\prime} }_{i}{A^{\prime} }_{j}}=8.275\pm 72$

**Table 12:** Experimental average values and theoretical results of observables for the input state $|{\overrightarrow{{\rm{e}}}}_{2})$. The dates behind the experimental average values in the parentheses are standard deviations.

Terms	Experimental value	Theoretical prediction	Terms	Experimental value	Theoretical prediction	Terms	Experimental value	Theoretical prediction
$\overline{{A^{\prime} }_{1}}$	0.325 (4)	0.333	$\overline{{A^{\prime} }_{1}{A^{\prime} }_{6}}$	−0.655 (15)	−0.667	$\overline{{A^{\prime} }_{5}{A^{\prime} }_{6}}$	−0.965 (0)	−1
$\overline{{A^{\prime} }_{2}}$	0.329 (19)	0.333	$\overline{{A^{\prime} }_{1}{A^{\prime} }_{7}}$	−0.676 (11)	−0.667	$\overline{{A^{\prime} }_{5}{A^{\prime} }_{11}}$	−0.025 (11)	0
$\overline{{A^{\prime} }_{3}}$	0.335 (6)	0.333	$\overline{{A^{\prime} }_{1}{A^{\prime} }_{9}}$	0.356 (5)	0.333	$\overline{{A^{\prime} }_{6}{A^{\prime} }_{11}}$	−0.010 (11)	0
$\overline{{A^{\prime} }_{4}}$	0.333 (4)	0.333	$\overline{{A^{\prime} }_{2}{A^{\prime} }_{5}}$	−0.667 (6)	−0.667	$\overline{{A^{\prime} }_{7}{A^{\prime} }_{8}}$	−0.961 (1)	−1
$\overline{{A^{\prime} }_{5}}$	0.010 (11)	0	$\overline{{A^{\prime} }_{2}{A^{\prime} }_{8}}$	−0.663 (1)	−0.667	$\overline{{A^{\prime} }_{7}{A^{\prime} }_{12}}$	−0.021 (16)	0
$\overline{{A^{\prime} }_{6}}$	0.025 (11)	0	$\overline{{A^{\prime} }_{2}{A^{\prime} }_{9}}$	0.364 (10)	0.333	$\overline{{A^{\prime} }_{8}{A^{\prime} }_{12}}$	−0.017 (17)	0
$\overline{{A^{\prime} }_{7}}$	0.017 (17)	0	$\overline{{A^{\prime} }_{3}{A^{\prime} }_{5}}$	−0.655 (9)	−0.667	$\overline{{A^{\prime} }_{9}{A^{\prime} }_{10}}$	0.960 (1)	1
$\overline{{A^{\prime} }_{8}}$	0.021 (16)	0	$\overline{{A^{\prime} }_{3}{A^{\prime} }_{7}}$	−0.667 (4)	−0.667	$\overline{{A^{\prime} }_{9}{A^{\prime} }_{13}}$	−0.969 (0)	−1
$\overline{{A^{\prime} }_{9}}$	0.992 (1)	1	$\overline{{A^{\prime} }_{3}{A^{\prime} }_{10}}$	0.313 (11)	0.333	$\overline{{A^{\prime} }_{10}{A^{\prime} }_{13}}$	−0.992 (1)	−1
$\overline{{A^{\prime} }_{10}}$	0.969 (0)	1	$\overline{{A^{\prime} }_{4}{A^{\prime} }_{6}}$	−0.664 (5)	−0.667	$\overline{{A^{\prime} }_{11}{A^{\prime} }_{12}}$	0.961 (1)	1
$\overline{{A^{\prime} }_{11}}$	0.992 (0)	1	$\overline{{A^{\prime} }_{4}{A^{\prime} }_{8}}$	−0.650 (11)	−0.667	$\overline{{A^{\prime} }_{11}{A^{\prime} }_{13}}$	−0.968 (1)	−1
$\overline{{A^{\prime} }_{12}}$	0.968 (1)	1	$\overline{{A^{\prime} }_{4}{A^{\prime} }_{10}}$	0.349 (15)	0.333	$\overline{{A^{\prime} }_{12}{A^{\prime} }_{13}}$	−0.992 (0)	−1
$\overline{{A^{\prime} }_{13}}$	−0.961 (1)	−1						
Original Yu-Oh	$\mathop{\sum }\limits_{i=1}^{13}\overline{{A^{\prime} }_{i}}-\frac{1}{4}\mathop{\sum }\limits_{i=1}^{13}\mathop{\sum }\limits_{j=1,j\ne i}^{13}{\Gamma }_{i,j}\overline{{A^{\prime} }_{i}{A^{\prime} }_{j}}=8.314\pm 86$

**Table 13:** Experimental average values and theoretical results of observables for the input state$\tfrac{1}{\sqrt{2}}(|{\overrightarrow{{\rm{e}}}}_{0})+|{\overrightarrow{{\rm{e}}}}_{1}))$. The dates behind the experimental average values in the parentheses are standard deviations.

Terms	Experimental value	Theoretical prediction	Terms	Experimental value	Theoretical prediction	Terms	Experimental value	Theoretical prediction
$\overline{{A^{\prime} }_{1}}$	0.953 (4)	1	$\overline{{A^{\prime} }_{1}{A^{\prime} }_{6}}$	0.408 (9)	0.5	$\overline{{A^{\prime} }_{5}{A^{\prime} }_{6}}$	0 (25)	0
$\overline{{A^{\prime} }_{2}}$	0.938 (1)	1	$\overline{{A^{\prime} }_{1}{A^{\prime} }_{7}}$	0.506 (1)	0.5	$\overline{{A^{\prime} }_{5}{A^{\prime} }_{11}}$	−0.499 (9)	−0.5
$\overline{{A^{\prime} }_{3}}$	−0.344 (5)	−0.333	$\overline{{A^{\prime} }_{1}{A^{\prime} }_{9}}$	−0.962 (2)	−1	$\overline{{A^{\prime} }_{6}{A^{\prime} }_{11}}$	−0.501 (21)	−0.5
$\overline{{A^{\prime} }_{4}}$	−0.375 (7)	−0.333	$\overline{{A^{\prime} }_{2}{A^{\prime} }_{5}}$	0.433 (14)	0.5	$\overline{{A^{\prime} }_{7}{A^{\prime} }_{8}}$	0.032 (7)	0
$\overline{{A^{\prime} }_{5}}$	0.501 (21)	0.5	$\overline{{A^{\prime} }_{2}{A^{\prime} }_{8}}$	0.468 (3)	0.5	$\overline{{A^{\prime} }_{7}{A^{\prime} }_{12}}$	−0.505 (5)	−0.5
$\overline{{A^{\prime} }_{6}}$	0.499 (9)	0.5	$\overline{{A^{\prime} }_{2}{A^{\prime} }_{9}}$	−0.968 (1)	−1	$\overline{{A^{\prime} }_{8}{A^{\prime} }_{12}}$	−0.527 (10)	−0.5
$\overline{{A^{\prime} }_{7}}$	0.527 (10)	0.5	$\overline{{A^{\prime} }_{3}{A^{\prime} }_{5}}$	−0.799 (3)	−0.833	$\overline{{A^{\prime} }_{9}{A^{\prime} }_{10}}$	−0.991 (0)	−1
$\overline{{A^{\prime} }_{8}}$	0.505 (5)	0.5	$\overline{{A^{\prime} }_{3}{A^{\prime} }_{7}}$	−0.826 (4)	−0.833	$\overline{{A^{\prime} }_{9}{A^{\prime} }_{13}}$	−0.917 (2)	−1
$\overline{{A^{\prime} }_{9}}$	−0.907 (2)	−1	$\overline{{A^{\prime} }_{3}{A^{\prime} }_{10}}$	−0.334 (12)	−0.333	$\overline{{A^{\prime} }_{10}{A^{\prime} }_{13}}$	0.907 (2)	1
$\overline{{A^{\prime} }_{10}}$	0.916 (2)	1	$\overline{{A^{\prime} }_{4}{A^{\prime} }_{6}}$	−0.823 (14)	−0.833	$\overline{{A^{\prime} }_{11}{A^{\prime} }_{12}}$	−0.990 (0)	−1
$\overline{{A^{\prime} }_{11}}$	0 (9)	0	$\overline{{A^{\prime} }_{4}{A^{\prime} }_{8}}$	−0.836 (11)	−0.833	$\overline{{A^{\prime} }_{11}{A^{\prime} }_{13}}$	−0.010 (9)	0
$\overline{{A^{\prime} }_{12}}$	0.010 (9)	0	$\overline{{A^{\prime} }_{4}{A^{\prime} }_{10}}$	−0.334 (11)	−0.333	$\overline{{A^{\prime} }_{12}{A^{\prime} }_{13}}$	0 (9)	0
$\overline{{A^{\prime} }_{13}}$	0.990 (0)	1						
Original Yu-Oh	$\mathop{\sum }\limits_{i=1}^{13}\overline{{A^{\prime} }_{i}}-\frac{1}{4}\mathop{\sum }\limits_{i=1}^{13}\mathop{\sum }\limits_{j=1,j\ne i}^{13}{\Gamma }_{i,j}\overline{{A^{\prime} }_{i}{A^{\prime} }_{j}}=8.246\pm 86$

**Table 14:** Experimental average values and theoretical results of observables for the input state $\tfrac{1}{\sqrt{2}}(|{\overrightarrow{{\rm{e}}}}_{0})+|{\overrightarrow{{\rm{e}}}}_{2}))$. The dates behind the experimental average values in the parentheses are standard deviations.

Terms	Experimental value	Theoretical prediction	Terms	Experimental value	Theoretical prediction	Terms	Experimental value	Theoretical prediction
$\overline{{A^{\prime} }_{1}}$	0.939 (1)	1	$\overline{{A^{\prime} }_{1}{A^{\prime} }_{6}}$	0.475 (7)	0.5	$\overline{{A^{\prime} }_{5}{A^{\prime} }_{6}}$	−0.008 (3)	0
$\overline{{A^{\prime} }_{2}}$	−0.343 (14)	−0.333	$\overline{{A^{\prime} }_{1}{A^{\prime} }_{7}}$	−0.953 (1)	−1	$\overline{{A^{\prime} }_{5}{A^{\prime} }_{11}}$	−0.498 (1)	−0.5
$\overline{{A^{\prime} }_{3}}$	0.951 (6)	1	$\overline{{A^{\prime} }_{1}{A^{\prime} }_{9}}$	0.483 (11)	0.5	$\overline{{A^{\prime} }_{6}{A^{\prime} }_{11}}$	−0.495 (5)	−0.5
$\overline{{A^{\prime} }_{4}}$	−0.330 (24)	−0.333	$\overline{{A^{\prime} }_{2}{A^{\prime} }_{5}}$	−0.823 (4)	−0.833	$\overline{{A^{\prime} }_{7}{A^{\prime} }_{8}}$	−0.997 (0)	−1
$\overline{{A^{\prime} }_{5}}$	0.495 (5)	0.5	$\overline{{A^{\prime} }_{2}{A^{\prime} }_{8}}$	−0.339 (5)	−0.333	$\overline{{A^{\prime} }_{7}{A^{\prime} }_{12}}$	−0.892 (4)	−1
$\overline{{A^{\prime} }_{6}}$	0.498 (1)	0.5	$\overline{{A^{\prime} }_{2}{A^{\prime} }_{9}}$	−0.826 (5)	−0.833	$\overline{{A^{\prime} }_{8}{A^{\prime} }_{12}}$	0.889 (4)	1
$\overline{{A^{\prime} }_{7}}$	−0.889 (4)	−1	$\overline{{A^{\prime} }_{3}{A^{\prime} }_{5}}$	0.499 (19)	0.5	$\overline{{A^{\prime} }_{9}{A^{\prime} }_{10}}$	−0.004 (2)	0
$\overline{{A^{\prime} }_{8}}$	0.892 (4)	1	$\overline{{A^{\prime} }_{3}{A^{\prime} }_{7}}$	−0.948 (5)	−1	$\overline{{A^{\prime} }_{9}{A^{\prime} }_{13}}$	−0.490 (3)	−0.5
$\overline{{A^{\prime} }_{9}}$	0.505 (3)	0.5	$\overline{{A^{\prime} }_{3}{A^{\prime} }_{10}}$	0.453 (6)	0.5	$\overline{{A^{\prime} }_{10}{A^{\prime} }_{13}}$	−0.505 (3)	−0.5
$\overline{{A^{\prime} }_{10}}$	0.490 (3)	0.5	$\overline{{A^{\prime} }_{4}{A^{\prime} }_{6}}$	−0.822 (6)	−0.833	$\overline{{A^{\prime} }_{11}{A^{\prime} }_{12}}$	−0.038 (3)	0
$\overline{{A^{\prime} }_{11}}$	0.017 (4)	0	$\overline{{A^{\prime} }_{4}{A^{\prime} }_{8}}$	−0.334 (1)	−0.333	$\overline{{A^{\prime} }_{11}{A^{\prime} }_{13}}$	−0.945 (0)	−1
$\overline{{A^{\prime} }_{12}}$	0.945 (0)	1	$\overline{{A^{\prime} }_{4}{A^{\prime} }_{10}}$	−0.827 (8)	−0.833	$\overline{{A^{\prime} }_{12}{A^{\prime} }_{13}}$	−0.017 (4)	0
$\overline{{A^{\prime} }_{13}}$	0.038 (3)	0						
Original Yu-Oh	$\mathop{\sum }\limits_{i=1}^{13}\overline{{A^{\prime} }_{i}}-\frac{1}{4}\mathop{\sum }\limits_{i=1}^{13}\mathop{\sum }\limits_{j=1,j\ne i}^{13}{\Gamma }_{i,j}\overline{{A^{\prime} }_{i}{A^{\prime} }_{j}}=8.189\pm 68$

**Table 15:** Experimental average values and theoretical results of observables for the input state $\tfrac{1}{\sqrt{2}}(|{\overrightarrow{{\rm{e}}}}_{1})+|{\overrightarrow{{\rm{e}}}}_{2}\,))$. The dates behind the experimental average values in the parentheses are standard deviations.

Terms	Experimental value	Theoretical prediction	Terms	Experimental value	Theoretical prediction	Terms	Experimental value	Theoretical prediction
$\overline{{A^{\prime} }_{1}}$	−0.355 (8)	−0.333	$\overline{{A^{\prime} }_{1}{A^{\prime} }_{6}}$	−0.342 (5)	−0.333	$\overline{{A^{\prime} }_{5}{A^{\prime} }_{6}}$	−0.985 (0)	−1
$\overline{{A^{\prime} }_{2}}$	0.871 (2)	1	$\overline{{A^{\prime} }_{1}{A^{\prime} }_{7}}$	−0.822 (4)	−0.833	$\overline{{A^{\prime} }_{5}{A^{\prime} }_{11}}$	−0.897 (5)	−1
$\overline{{A^{\prime} }_{3}}$	0.891 (1)	1	$\overline{{A^{\prime} }_{1}{A^{\prime} }_{9}}$	−0.799 (3)	−0.833	$\overline{{A^{\prime} }_{6}{A^{\prime} }_{11}}$	0.882 (5)	1
$\overline{{A^{\prime} }_{4}}$	−0.235 (8)	−0.333	$\overline{{A^{\prime} }_{2}{A^{\prime} }_{5}}$	−0.953 (1)	−1	$\overline{{A^{\prime} }_{7}{A^{\prime} }_{8}}$	−0.028 (10)	0
$\overline{{A^{\prime} }_{5}}$	−0.882 (5)	−1	$\overline{{A^{\prime} }_{2}{A^{\prime} }_{8}}$	0.427 (19)	0.5	$\overline{{A^{\prime} }_{7}{A^{\prime} }_{12}}$	−0.494 (11)	−0.5
$\overline{{A^{\prime} }_{6}}$	0.897 (5)	1	$\overline{{A^{\prime} }_{2}{A^{\prime} }_{9}}$	0.408 (8)	0.5	$\overline{{A^{\prime} }_{8}{A^{\prime} }_{12}}$	−0.479 (0)	−0.5
$\overline{{A^{\prime} }_{7}}$	0.479(0)	0.5	$\overline{{A^{\prime} }_{3}{A^{\prime} }_{5}}$	−0.959 (0)	−1	$\overline{{A^{\prime} }_{9}{A^{\prime} }_{10}}$	−0.017 (5)	0
$\overline{{A^{\prime} }_{8}}$	0.494 (11)	0.5	$\overline{{A^{\prime} }_{3}{A^{\prime} }_{7}}$	0.457 (3)	0.5	$\overline{{A^{\prime} }_{9}{A^{\prime} }_{13}}$	−0.504 (16)	−0.5
$\overline{{A^{\prime} }_{9}}$	0.479 (18)	0.5	$\overline{{A^{\prime} }_{3}{A^{\prime} }_{10}}$	0.434 (2)	0.5	$\overline{{A^{\prime} }_{10}{A^{\prime} }_{13}}$	−0.479 (18)	−0.5
$\overline{{A^{\prime} }_{10}}$	0.504 (16)	0.5	$\overline{{A^{\prime} }_{4}{A^{\prime} }_{6}}$	−0.339 (5)	−0.333	$\overline{{A^{\prime} }_{11}{A^{\prime} }_{12}}$	−0.005 (6)	0
$\overline{{A^{\prime} }_{11}}$	0.996 (1)	1	$\overline{{A^{\prime} }_{4}{A^{\prime} }_{8}}$	−0.816 (11)	−0.833	$\overline{{A^{\prime} }_{11}{A^{\prime} }_{13}}$	0 (7)	0
$\overline{{A^{\prime} }_{12}}$	0 (7)	0	$\overline{{A^{\prime} }_{4}{A^{\prime} }_{10}}$	−0.820 (5)	−0.833	$\overline{{A^{\prime} }_{12}{A^{\prime} }_{13}}$	−0.996 (1)	−1
$\overline{{A^{\prime} }_{13}}$	0.005 (6)	0						
Original Yu-Oh	$\mathop{\sum }\limits_{i=1}^{13}\overline{{A^{\prime} }_{i}}-\frac{1}{4}\mathop{\sum }\limits_{i=1}^{13}\mathop{\sum }\limits_{j=1,j\ne i}^{13}{\Gamma }_{i,j}\overline{{A^{\prime} }_{i}{A^{\prime} }_{j}}=8.205\pm 82$							
